# Self-face Captures, Holds, and Biases Attention

**DOI:** 10.3389/fpsyg.2017.02371

**Published:** 2018-01-11

**Authors:** Michał J. Wójcik, Maria M. Nowicka, Ilona Kotlewska, Anna Nowicka

**Affiliations:** ^1^Laboratory of Psychophysiology, Department of Neurophysiology, Nencki Institute of Experimental Biology, Warsaw, Poland; ^2^Wroclaw Faculty of Psychology, SWPS University of Social Sciences and Humanities, Wroclaw, Poland; ^3^Faculty of Humanities, Nicolaus Copernicus University, Torun, Poland

**Keywords:** self-recognition, self-face, attention, ERP, attentional bias, N2pc, SPCN

## Abstract

The implicit self-recognition process may take place already in the pre-attentive stages of perception. After a silent stimulus has captured attention, it is passed on to the attentive stage where it can affect decision making and responding. Numerous studies show that the presence of self-referential information affects almost every cognitive level. These effects may share a common and fundamental basis in an attentional mechanism, conceptualized as attentional bias: the exaggerated deployment of attentional resources to a salient stimulus. A gold standard in attentional bias research is the dot-probe paradigm. In this task, a prominent stimulus (cue) and a neutral stimulus are presented in different spatial locations, followed by the presentation of a target. In the current study we aimed at investigating whether the self-face captures, holds and biases attention when presented as a task-irrelevant stimulus. In two dot-probe experiments coupled with the event-related potential (ERP) technique we analyzed the following relevant ERPs components: N2pc and SPCN which reflect attentional shifts and the maintenance of attention, respectively. An inter-stimulus interval separating face-cues and probes (800 ms) was introduced only in the first experiment. In line with our predictions, in Experiment 1 the self-face elicited the N2pc and the SPCN component. In Experiment 2 in addition to N2pc, an attentional bias was observed. Our results indicate that unintentional self-face processing disables the top-down control setting to filter out distractors, thus leading to the engagement of attentional resources and visual short-term memory.

## Introduction

Although being yourself is a profound and undeniable belief, it is not obvious from a functional perspective. The unconscious machinery that is the cornerstone of the self works perpetually in order to recognize as well as consolidate different and temporally separated pieces of self-related information into one coherent whole (McAdams, 2001; Conway, 2005). Without it, there would be no concept of “me” as distinct from “you,” no self-conscious thought, no identity.

The ability to cognitively identify oneself as an object in the environment, which is self-recognition (Platek et al., 2004), is the central process that enables maintaining the coherence of the self. It can be described on two levels: (1) implicit as the preference of self-related information, and (2) explicit, as identification of one's own image (Ross et al., 2011). As the Self Attention Network model states, the implicit self-recognition process may take place already in the pre-attentive stages of perception, assigning a saliency property to self-related information that includes the image of one's own face (Humphreys and Sui, 2016).

Existing evidence related to the attentional capture effect of self-face stimuli is rather inconclusive. For example, Tong and Nakayama (1999) showed that the self-face was detected faster among distractors than an other-face even if presented in an unusual orientation. An attention-grabbing property of the self-face was also shown by Brédart et al. (2006). A flanking self-face caused a stronger interference in the detection of a classmate's name in comparison to the reversed condition. In contrast, Devue et al. (2009) reported that if the self-face was presented outside of the participant's focus of attention, it failed in capturing attention.

Theeuwes (2010), as well as Itti and Koch (2001), have proposed that after a silent stimulus has captured attention, it is passed on to the attentive stage where it is identified. Because the information about this stimulus is now available for the top-down processes, it can affect decision making and responding. Numerous studies show that the presence of self-referential information affects almost every cognitive level (Humphreys and Sui, 2016). Self-related information alters perception (Sui et al., 2015), attention (Tong and Nakayama, 1999; Brédart et al., 2006; Devue et al., 2009), memory (Symons and Johnson, 1997) and even meta-cognition (Pronin et al., 2002). Moreover, self-bias exerts influence also on social perception (Ross et al., 1977).

One may suppose that these effects share a common and fundamental basis in an attentional mechanism described by Theeuwes (2010), which could be conceptualized as attentional bias. This term refers to the tendency for people's perception to be affected by previously processed information (Bar-Haim et al., 2007). It relies on the exaggerated deployment of attentional resources to a salient stimulus that is present in a person's external environment.

A gold standard in attentional bias research is the dot-probe paradigm, which also enables investigation of attentional capture effects (Pfabigan and Tran, 2015). In this task, a prominent stimulus (cue) and a neutral stimulus are presented at the same time in different spatial locations (e.g., one to the left and one to the right of the central fixation point), followed by the presentation of a target at cued or not cued locations. Reaction times (RTs) to targets that appear at the prior location of the prominent stimulus (i.e., cue-congruent trials) are compared with RTs to targets that appear at the prior location of the neutral stimulus (i.e., cue-incongruent trials). Faster responses typically observed in cue-congruent trials are interpreted as evidence of an attentional bias to the location of the prominent stimulus.

In the current study, we aimed at investigating whether the self-face captures, holds and biases attention when presented as a task-irrelevant, to-be-ignored stimulus. To achieve these goals, we conducted two experiments using the dot-probe task coupled with electrophysiological measurements (EEG). Both of them were intended to reveal attention-grabbing properties of self-face as operationalized by the emergence of a lateralized event-related potentials (ERPs) component N2-posterior-contralateral, N2pc (Eimer and Kiss, 2008; Sawaki and Luck, 2010). The presence of attentional hold effects can be, in turn, evidenced by a sustained posterior contralateral negativity component (SPCN). Because this component reflects later stages of information processing, a prolonged time of cue presentation and delay between the cue and target onsets were introduced in Experiment 1. Finally, faster responses to targets preceded by self-face in comparison to targets appearing contralateral to the self-face will indicate an attentional bias (Experiment 2).

The N2pc component consists of a greater negativity at the contralateral sites than the ipsilateral sites to an attended stimuli. It is typically detected at posterior scalp sites, approximately 200–300 ms after stimulus onset, with a maximum voltage at the parietal-occipital region (Luck and Hillyard, 1994; Eimer, 1996). N2pc is used to determine whether the focus of attention has covertly been shifted to the location of a silent stimulus. It reflects the allocation of a limited-capacity process to a relevant object (Ester et al., 2012). Previous dot-probe studies reported the presence of N2pc for prominent stimuli such as emotional faces (Holmes et al., 2009; Grimshaw et al., 2014). To the best of our knowledge, none of the studies used the self-face as N2pc-evoking stimulus.

In tasks that engage visual short-term memory (Jolicœur et al., 2008) an SCPN component is often observed. It begins about 300–400 ms after stimulus onset and persists for the duration of the retention interval. The SPCN is also present in tasks that are not defined as memory tasks, but that engage visual short-term memory as an intermediate processing buffer (Jolicœur et al., 2008). This component is computed as the difference between contralateral and ipsilateral activity time-locked to a lateralized stimulus. Its amplitude increases with visual working memory (VWM) informational load (e.g., Vogel and Machizawa, 2004; Jolicœur et al., 2008; Perron et al., 2009; Robitaille et al., 2009) and with increased number (Vogel and Machizawa, 2004) and the complexity (Luria et al., 2010) of stimuli to be held in VWM. Sessa et al. (2011, 2012) have further demonstrated that SPCN amplitude is modulated by emotional expressions of faces and varies proportionally to the resolution of the faces' representations in VWM, such that representations of high-resolution faces elicit larger SPCN amplitudes relative to representations of low-resolution faces.

It is worth noting that biases caused by different cue presentation times reflect biases in different stages of processing (Cisler and Koster, 2010). Longer cue durations may lead to the difficulty in disengaging attention from prominent stimuli, which may be caused by the failure of the dorsal fronto-parietal network to control the deployment of attention (Corbetta and Shulman, 2002; De Raedt and Koster, 2010). In turn, shorter cue duration times (typically less than 500 ms) reflect rapid orienting to the silent stimulus. This exaggerated sensitivity is caused by a stimulus-driven detection mechanism that is likely to involve the amygdala, accompanied by a failure of control mechanisms in the left lateral prefrontal cortex, which normally filters out the to-be-ignored stimulus (Bishop et al., 2004, 2007). Therefore, we hypothesized that the extended cue presentation time in Experiment 1 should produce an SPCN component. In order to prevent the influence of ongoing perceptual processing on this component, we separated the self-face cue from the target with a black-screen. It should be clarified that this inter-stimulus interval reduces the influence of the cue on the target, thus probably eliminating the facilitation effect regarding reaction times. In Experiment 2 we focused on investigating the attentional bias toward the self-face, thus the cue and the target were not separated.

## Method

### Participants

Twenty-one subjects (10 female) between the ages of 24 and 35 (*M* = 27.6, *SD* = 3) participated in the study. All participants had normal or corrected-to-normal vision and reported no history of mental or neurological disorders. Twenty of these subjects were right-handed, as assessed by the Edinburgh Handedness Inventory (Oldfield, 1971). The study was conducted with the approval of the Human Ethics Committee of the SWPS University of Social Sciences and Humanities (Warsaw, Poland). All participants gave written informed consent prior to the experiments.

### Procedure and apparatus

After electrode cap placement (ActiCAP, Brain Products, Munich, Germany), participants were seated in a comfortable chair in a dimly lit and sound-attenuated room. During the task, an adjustable chin rest maintained head position and a constant viewing distance of 72 cm. The dot-probe task was presented on a Flex Scan EV-2450 (Hakusan, Ishikawa, Japan) computer screen through an Intel Core i3 computer running Presentation® software (Neurobehavioral Systems, Albany, CA, USA). The screen was specially calibrated for correction to black in order to not exhaust eyes with intense background illumination. EEG signal was amplified using QuickAmp and digitized using BrainVision Recorder® software (Brain Products, Munich, Germany).

### Stimuli

**Figure 1:**
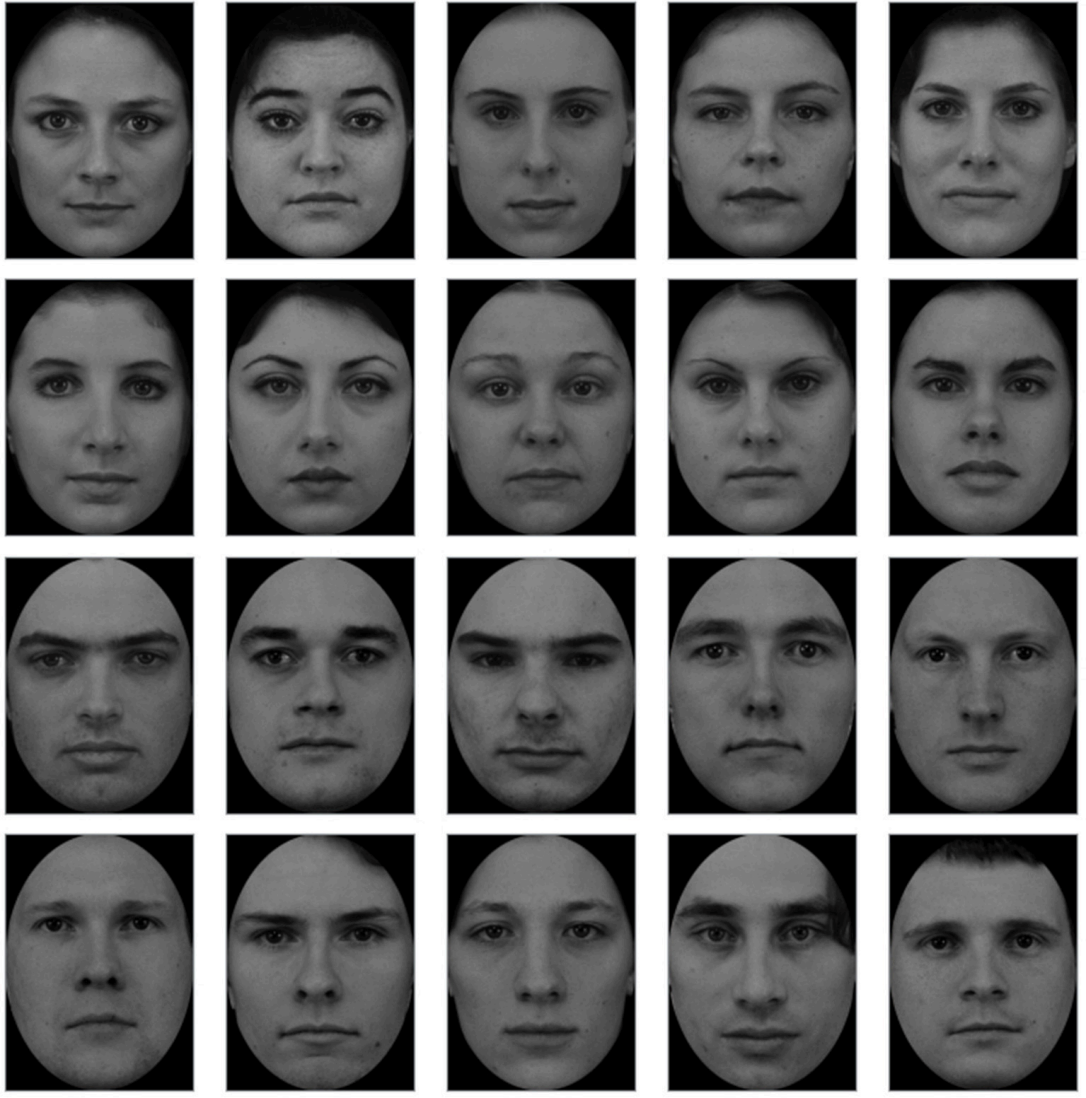
Set of example other-faces stimuli (F01, F03, F05, F07, F13, F23, F24, F26, F29 and M01, M06, M07, M08, M10, M11, M14, M18, M25, M31) taken from the A series of the Karolinska Directed Emotional Faces database (Lundqvist et al., 1998).

The stimuli consisted of bilaterally presented pairs of gray-scaled face photographs. Depending on the condition, stimuli pairs contained either a self-face and an other-face or two other-faces. Twenty-six (13 male and 13 female) other-face photographs were taken from the A series of the Karolinska Directed Emotional Faces database (Lundqvist et al., 1998). Figure 1 presents example stimuli used in the current study. In order to avoid effects of facial expressions, the photos were selected based on the unbiased hit-rates for neutral expression detection (Goeleven et al., 2008). Self-face photographs were taken prior to the experiments. All stimuli were cropped to include only the face, resized to subtend 6.9° × 8.9° of visual angle and equaled for mean luminance using Photoshop® (Adobe, San Jose, CA). Cue-faces appeared on the screen with their inner edge 3° left and right from the fixation. This distance is sufficient to detect horizontal eye movements and, as a consequence, to reject trials contaminated with these artifacts (Meyberg et al., 2017). The gender of other-faces was matched to each participant's gender in order to control the between-category variability in attentional effects.

### Dot-probe task

#### Experiment 1

**Figure 2:**
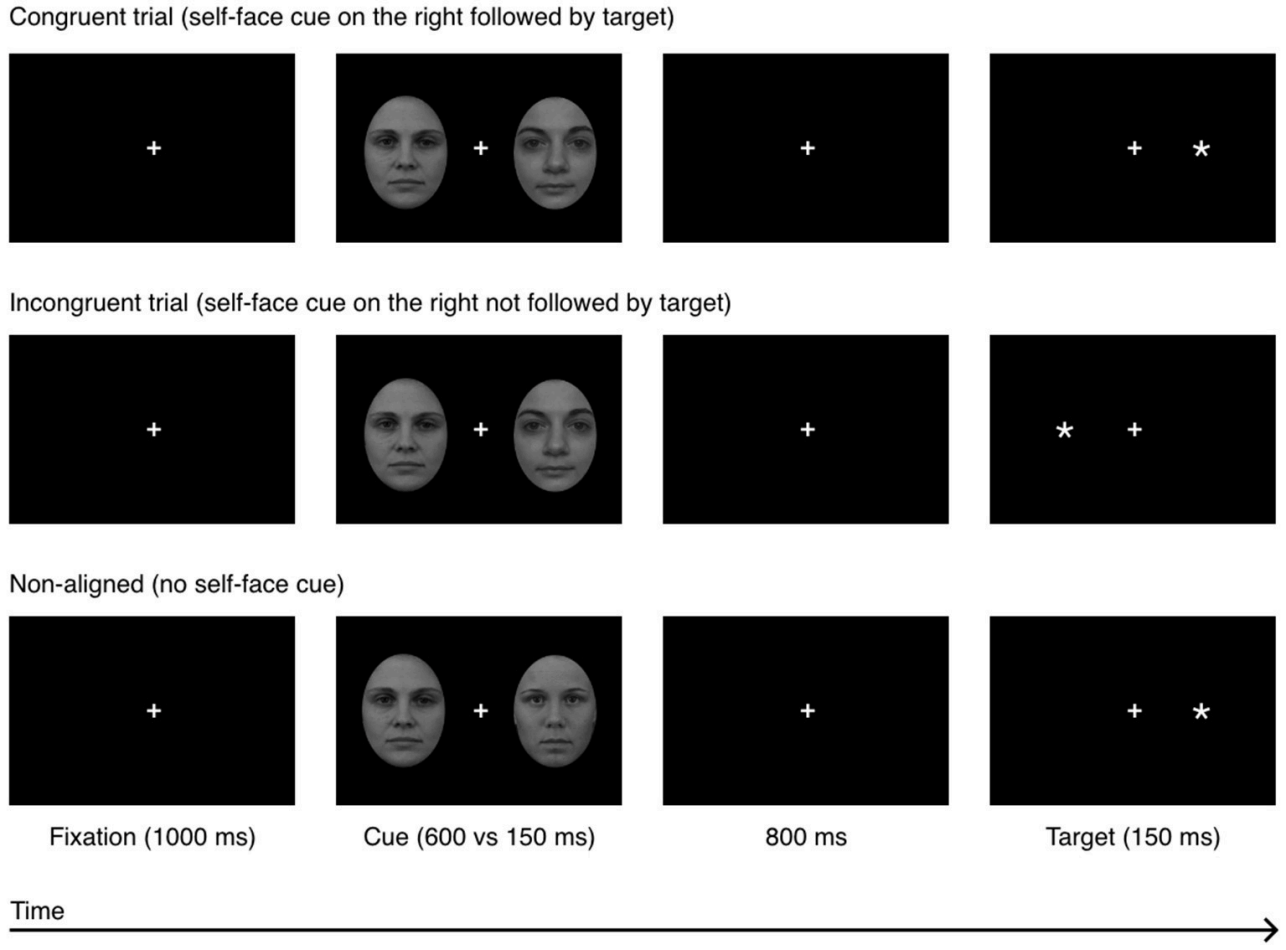
Experiment 1: a sequence of events in a single experimental trial in 3 experimental conditions: congruent, incongruent, and non-aligned. The example self-face is a photograph of one of the co-authors; other-faces stimuli (F19 and F34) were taken from the A series of the Karolinska Directed Emotional Faces database (Lundqvist et al., 1998).

Each trial started with a fixation cross (subtending 0.4° × 0.4° of visual angle; positioned in the center of the screen) which remained onscreen for the duration of the trial. After 1,000 ms, a pair of faces was presented bilaterally. In order to minimalize the occurrence of readiness potentials, the face-cues were presented for either 150 or 600 ms (Libet et al., 1983). Cues were followed by a black-screen which remained on screen for 800 ms. The probe (an asterisk subtending 0.3° × 0.3° of visual angle) was then displayed in the visual field previously occupied by the self-face (congruent condition), in the opposed visual field (incongruent condition) or in the visual field previously occupied by one of two bilaterally presented other-faces, i.e., non-aligned condition (see Figure 2). In the first two conditions, an other-face was presented contralateral to the self-face. Then the participants were instructed to indicate (as quickly and as accurately as possible) the side on which the probe appeared, by pressing with their left or right index finger the button corresponding to the probe's location. The participants were requested to maintain fixation on the cross and ignore the face cues.

#### Experiment 2

**Figure 3:**
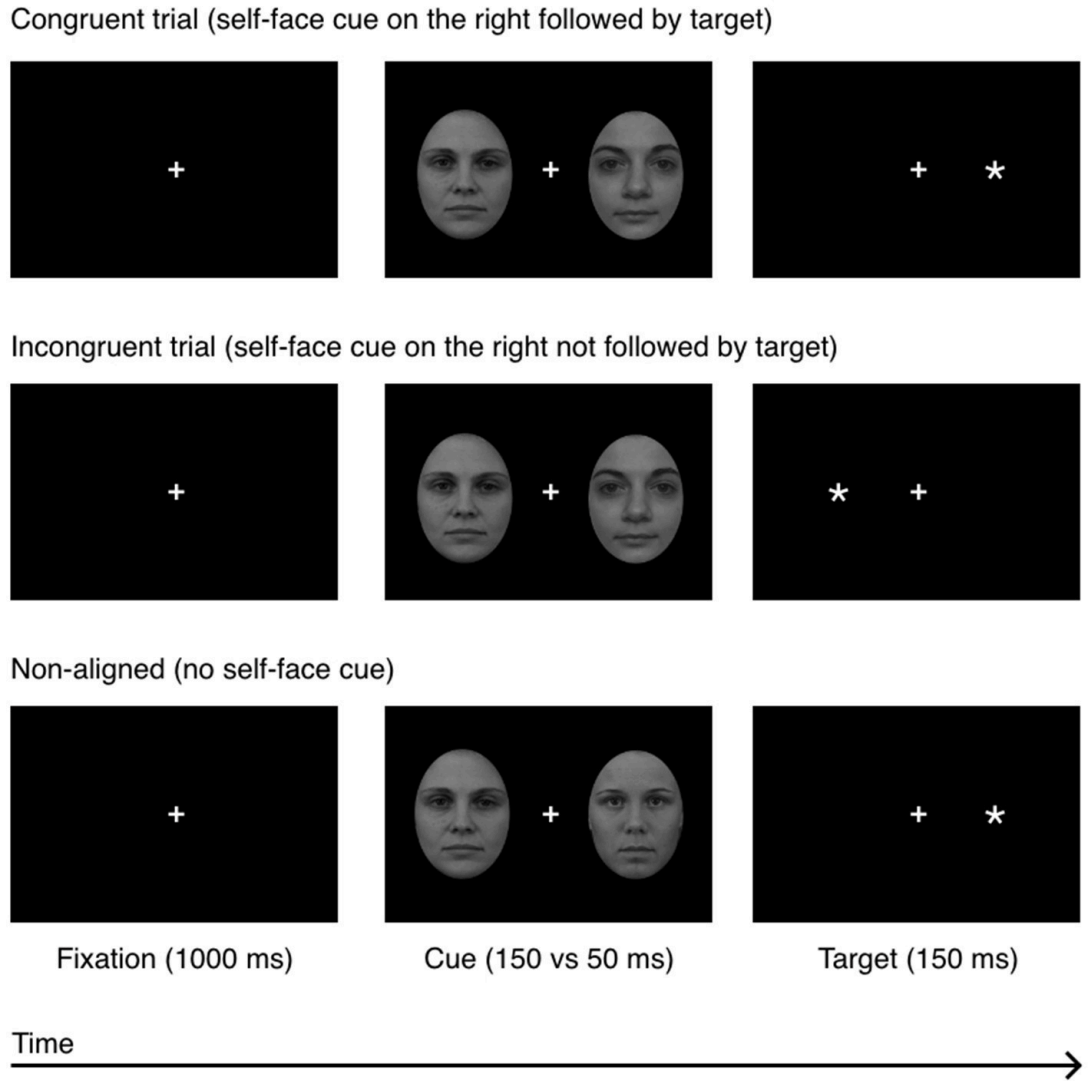
Experiment 2: a sequence of events in a single experimental trial in 3 experimental conditions: congruent, incongruent, and non-aligned. The example self-face is a photograph of one of the co-authors; other-faces stimuli (F19 and F34) were taken from the A series of the Karolinska Directed Emotional Faces database (Lundqvist et al., 1998).

The face-cues were presented for 50 and 150 ms and were not separated from the probe by a black-screen. All other details were the same as in Experiment 1. The experimental procedure is shown in Figure 3.

### EEG and EOG data

The electroencephalogram (EEG) was continuously recorded with 64 Ag-AgCl electrically shielded electrodes mounted on an elastic cap (ActiCAP, Munich, Germany) and positioned according to the extended 10–20 system. For ocular artifact scoring, vertical and horizontal electrooculograms (EOGs) were recorded from bipolar electrodes placed at the supra- and suborbit of the right eye and at external canthi of eyes. EEG electrode impedances were kept below 10 kΩ. The data were amplified using a 128-channel amplifier (QuickAmp, Brain Products, Enschede, Netherlands) and digitized at a 500 Hz sampling rate. The EEG signal was recorded against an average of all channels calculated by the amplifier hardware. Offline, the 62 channels were re-referenced to the algebraic average of the left and right earlobes, notch filtered at 50 Hz and digitally band-pass filtered from 1 to 30 Hz using a zero-phase Butterworth filter (12 dB/oct).

### ERP analysis

**Figure 4:**
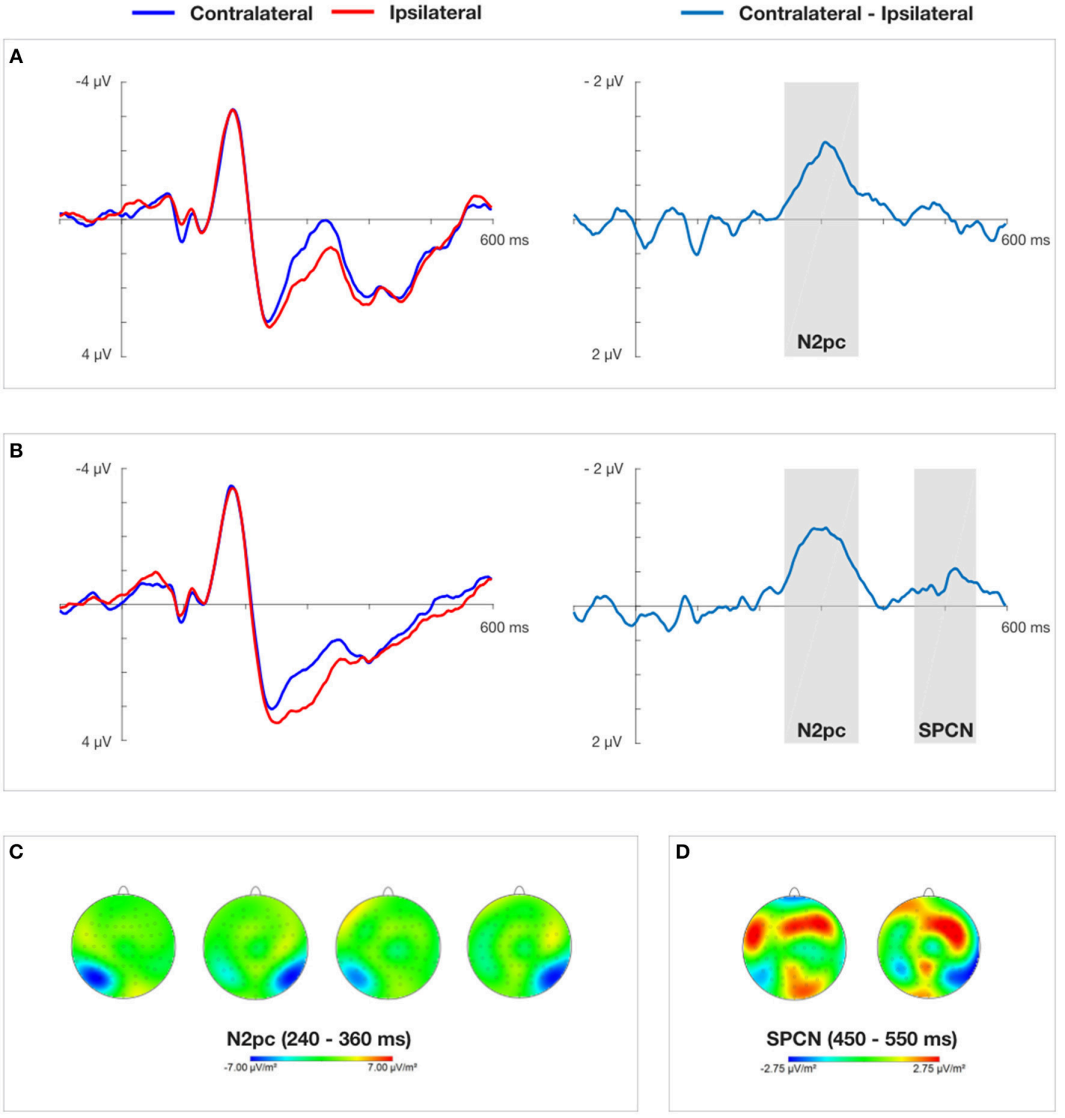
Grand average ERPs time-locked to the onset of the cue-faces (self vs. other) at electrodes PO7/PO8 and difference waves for 150 ms **(A)** and 600 ms **(B)** cue presentation time obtained in Experiment 1. **(C)** Presents topographical CSD maps of activity distribution in N2pc time window obtained by subtracting the non-aligned condition from the conditions containing cues. Two maps on the left (150 ms cue presentation) and two maps on the right (600 ms cue presentation) show amplitude distributions for self-face presented in the right and left visual field, respectively. **(D)** Presents topographical CSD maps of activity distribution in SPCN time window obtained by subtracting the non-aligned condition from the conditions containing cues. The map on the left and the right show amplitude distributions for self-face presented in the right and left visual field, respectively.

**Figure 5:**
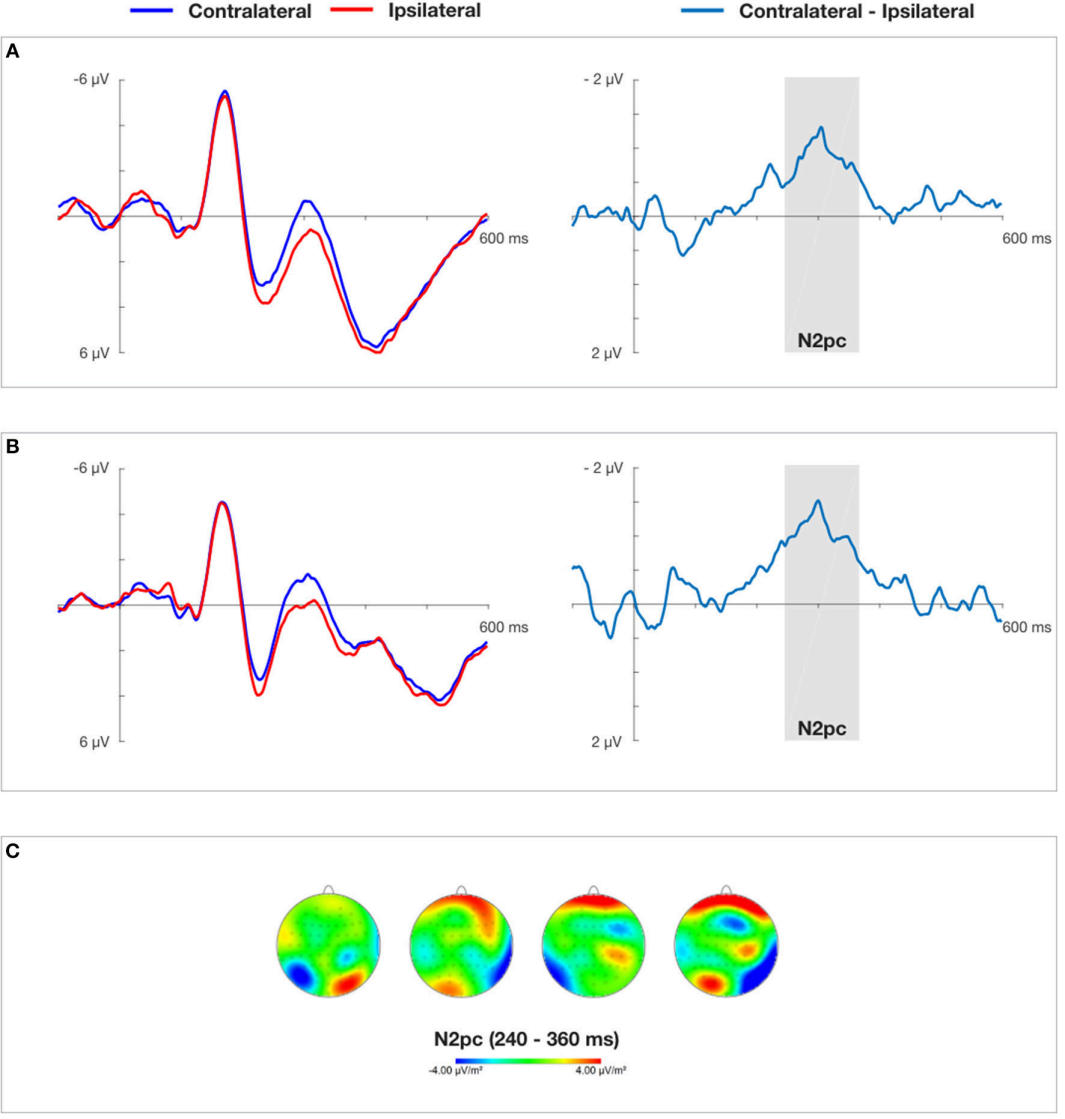
Grand average ERPs time-locked to the onset of the cue-faces (self vs. other) at electrodes PO7/PO8 and difference waves for 50 ms **(A)** and 150 ms **(B)** cue presentation time obtained in Experiment 2. **(C)** Presents topographical CSD maps of activity distribution in N2pc time window obtained by subtracting the non-aligned condition from the conditions containing cues. Two maps on the left (50 ms cue presentation) and two maps on the right (150 ms cue presentation) show amplitude distributions for self-face presented in the right and left visual field, respectively.

Occipital-temporal channels PO8 and PO7 were chosen for the ERPs analysis. These electrodes are frequently reported by many authors as disclosing maximal N2pc amplitudes (Eimer and Kiss, 2008; Jolicœur et al., 2008; Burra and Kerzel, 2013). Moreover, CSD topographic maps (obtained by subtracting non-aligned conditions from those containing the self-cue) showed a clear distribution of N2pc that is maximal over the parietal-occipital region (see Figures 4, 5). The EEG signal was segmented into 800 ms epochs from 200 ms before to 600 after cue onset. These epochs were baseline-corrected against the mean voltage during the 200 ms pre-stimulus period. Epochs contaminated with vertical eye movements and blinks (a change in voltage in the VEOG channel exceeding 100 μV within a 200 ms period), horizontal eye movements (a change in voltage in the HEOG channel exceeding 50 μV within any 200 ms period) or other artifacts (in all channels: voltage steps exceeding 50 μV, voltage change exceeding 100 μV within any 200 ms period, amplitudes greater than 200 μV and lower than −200 μV, activity in 100 ms intervals lower than 0.5 μV) were rejected. Because of excessive artifact contamination, three participants were excluded from the analyses (82.5, 49.2, and 48% rejected trials) in Experiment 1 and three (93.8, 84.4, and 63.1% rejected trials) in Experiment 2. An average of 15 and 20.5% of trials per subject was rejected in Experiment 1 and Experiment 2, respectively. The mean number of segments in Experiment 1 which passed the artifact rejection procedure was as follows: 600 ms cue presentation time (*M* = 138, *SD* = 14), 150 ms cue presentation time (*M* = 136, *SD* = 16). The mean number of segments in Experiment 2 which passed the artifact rejection procedure was as follows: 150 ms cue presentation time (*M* = 61, *SD* = 14), 50 ms cue presentation time (*M* = 66, *SD* = 12). The number of epochs used to compute ERPs did not differ significantly between conditions (ipsilateral and contralateral waveforms were computed based on the same datasets).

The ipsilateral waveform was calculated as the average of signals recorded at PO7 electrode to the left-sided self-face stimulus and at PO8 electrode to the right-sided self-face stimulus. The contralateral waveform was computed as the average of signals recorded at PO7 electrode to the right-sided self-face stimulus and at PO8 electrode to the left-sided self-face stimulus. In order to clearly visualize the N2pc and SPCN components and isolate them from overlapping components a difference between collapsed contralateral and ipsilateral waveforms was calculated (Luck, 2014).

On the basis of visual inspection of grand average contralateral-ipsilateral waveforms obtained in Experiment 1 and Experiment 2 (see Figures 4, 5), mean amplitude in 240–360 ms time window was chosen for successive statistical analysis. These quantifications of the N2pc component are in line with previous studies on electrophysiological markers of attentional capture (Eimer and Kiss, 2007; Buodo et al., 2009; Holmes et al., 2009; Grimshaw et al., 2014). After the contralateral and ipsilateral curves converged in Experiment 1, indicating the end of the N2pc component, another contralateral negativity emerged, corresponding to the onset of the SPCN component (Jolicœur et al., 2008). It was analyzed in 450–550 ms time window. Non-aligned trials were not taken into account in this analysis, as they lacked a laterally-located cue that could serve as a reference.

## Results

### Experiment 1

#### Behavioral analyses

All trials with RTs shorter than 100 ms and longer than 1,000 ms were excluded from analysis. Mean RTs for correct trials were computed for each participant for each condition (see Table 1 for descriptive statistics). The same three subjects as in the ERP analysis were excluded from the analysis because of artifact contamination. A three (condition: congruent vs. incongruent vs. non-aligned) × two (cue presentation time: 150 ms vs. 600 ms) repeated measures analysis of variance was computed. A main effect of “cue presentation time” was found, *F*_(1, 17)_ = 27.738, *p* < 0.000, η_*p*_^2^ = 0.620, showing that the subjects were faster in response when the self-face was presented for 600 ms (*M* = 370.5 ms) than for 150 ms (*M* = 383.4 ms). Other effects and interactions were non-significant.

**Table 1:** Descriptive statistics of behavioral results in Experiment 1.

**Cue presentation time**	**Condition**	**Mean**	***SD***	***N***
600 ms	Congruent	370.2	48.20	18
	Incongruent	370.2	49.80	18
	Non-aligned	371.3	51.60	18
150 ms	Congruent	384.8	54.29	18
	Incongruent	385.8	55.25	18
	Non-aligned	379.7	52.21	18

#### N2pc

A two (laterality: contralateral vs. ipsilateral) × two (cue presentation time: 150 ms vs. 600 ms) repeated measures ANOVA yielded a significant main effect of laterality, *F*_(1, 17)_ = 17.618, *p* < 0.001, η_*p*_^2^ = 0.509. This indicates a clear N2pc for both cue presentation times quantified as more negative mean amplitudes of the contralateral (*M* = 1.455 μV) than the ipsilateral (*M* = 2.242 μV) waveform. The main effect of “cue presentation time” also reached significance, *F*_(1, 17)_ = 16.835, *p* < 0.001, η_*p*_^2^ = 0.498, showing more negative amplitudes for 150 ms than 600 ms of self-face presentation. The interaction between these two factors was non-significant.

#### SPCN

The repeated measures ANOVA performed on “laterality” and “cue presentation time” yielded a significant interaction between these two factors, *F*_(1, 17)_ = 9.424, *p* < 0.05, η_*p*_^2^ = 0.357, indicating that an SPCN was only present when the cue was displayed for 600 ms. The factor of “cue presentation time” also obtained a significant main effect, *F*_(1, 17)_ = 5.320, *p* < 0.05, η_*p*_^2^ = 0.238, showing more negative amplitudes for 600 ms than 150 ms of cue presentation time.

### Experiment 2

#### Behavioral analyses

Based on the mean percentage of trials contaminated with ocular artifacts per participant, the same three participants as in the ERP analysis were excluded from the sample. Descriptive statistics regarding every experimental condition are presented in Table 2. The data structure was analyzed with a three (condition: congruent vs. incongruent vs. non-aligned) × two (cue presentation time: 150 ms vs. 50 ms) repeated measures ANOVA. The main effect of “condition,” *F*_(2, 34)_ = 3.996, *p* < 0.05, η_*p*_^2^ = 0.190, indicated a significant difference in reaction times. *Post-hoc* tests revealed that overall participants were faster in response when the target appeared in the visual field previously occupied by the self-face (*M* = 368.9 ms) than in the incongruent condition (*M* = 375.6 ms), *p*_*b*_ < 0.05. The main effect of “cue presentation time” also reached significance, *F*_(1, 17)_ = 18.915, *p* < 0.001, $\eta_{p}^{2}$ = 0.527, showing faster responses in trials with cues displayed for 150 ms (363.7 ms) than 50 ms (380.8 ms). The interaction between the aforementioned factors was non-significant.

**Table 2:** Descriptive statistics of behavioral results in Experiment 2.

**Cue presentation time**	**Condition**	**Mean**	***SD***	***N***
150 ms	Congruent	359.1	38.37	18
	Incongruent	367.3	38.05	18
	Non-aligned	364.8	35.47	18
50 ms	Congruent	378.8	36.13	18
	Incongruent	384.0	34.54	18
	Non-aligned	379.7	32.12	18

#### N2pc

In order to assess the presence of the N2pc component a two (laterality: contralateral vs. ipsilateral) × two (cue presentation time: 150 ms vs. 50 ms) repeated measures analysis of variance was performed. It yielded a significant main effect of laterality, *F*_(1, 17)_ = 28.552, *p* < 0.001, η_*p*_^2^ = 0.627, indicating that overall the mean amplitudes of the contralateral waveform were more negative (*M* = 0.423 μV), than the mean amplitudes of the ipsilateral waveform (*M* = 1.397 μV). The main effect of “cue presentation time” also reached significance, *F*_(1, 17)_ = 7.916, *p* < 0.05, $\eta_{p}^{2}$ = 0.318, showing more negative amplitudes for 50 ms than 150 ms of self-face presentation (see Figure 5). The interaction between these two factors was non-significant.

## Discussion

The aim of the current study was to examine whether the self-face captures automatic attention and as a consequence produces an attentional bias. Additionally, we assessed the attentional hold effects of this stimulus. The time course of the attentional effects was investigated using the ERP technique, which allowed us to trace the neural basis and the time-course of cognitive processes that occur very fast. Our analysis was focused on two ERPs components, N2pc and SPCN which reflect attentional shift and the engagement of visual short-term memory, respectively. On the behavioral level, attentional bias was evidenced by comparing RTs to targets preceded by self-face in comparison to RTs to targets preceded by other-faces. In general, the findings of this study supported all our predictions.

In line with our predictions, the self-face elicited a clear N2pc component in both dot-probe tasks: with and without a time delay between onsets of the cue (involving bottom-up processes) and target (involving top-down processes). The emergence of N2pc reflects the self-face's attention-grabbing properties on a neural level. Previous ERP studies on self-face recognition suggested such enhanced attentional processing; however, without specifying the stage of processing in which this effect occurs (Sui et al., 2006; Tacikowski and Nowicka, 2010). The only study—to our best knowledge—that did not confirm prioritized visual selection of the self-face was a behavioral study of (Devue et al., 2009). Such discrepancy may be related to the issue of parallel vs. serial processing. It seems that bottom-up processes in the visual search paradigm applied by Devue and colleagues took place parallel to the ongoing top-down processing. Due to the attentional bottleneck that occurs at the advanced stages of the top-down processing (indicated by the delayed time to arrive at the self-face), the self-preference effects could be inhibited (Sigman and Dehaene, 2008). This was not the case in the present study as all visual stimuli (cue and probe) were processed in a serial manner.

An important and novel finding of the present dot-probe study is the attentional bias toward self-face, whereas previous studies reported such bias only for emotional faces, especially threatening ones (Bar-Haim et al., 2007). The facilitation effect (faster RTs) observed in our study may result from the enhanced neural sensitivity at locations that were previously in the focus of attention. This notion is based on findings showing that directing attention to a specific area of the visual field enhances contrast sensitivity and spatial resolution at the attended location (Carrasco et al., 2004). Moreover, this attentional bias may be caused by a difficulty in disengaging attention from self-face, a phenomenon that was reported by Devue et al. (2009). These two interpretations may even complement each other.

Another unique feature of the present study is the presence of SPCN that was found exclusively for longer (600 ms) self-face cue presentations, indicating an attentional hold effect. This component has been typically observed when the task required encoding the peripheral stimulus and is associated with visual short-term memory (Jolicœur et al., 2008). Taking into account that the self-face could be viewed as a silent, prominent stimulus, the emergence of SPCN in our study is in line with an EEG study that analyzed this component for emotional faces (Stout et al., 2013). Despite the fact that in the present study cues were supposed to be ignored by participants a clear SPCN emerged. This indicates that unintentional self-face processing disables the top-down control setting to filter out distractors, thus leading to the engagement of visual short-term memory. Apparently, the visual system treats self-referential stimuli as prioritized, thus neglecting their status as distractors. As Holmes et al. (2009) propose, SPCN may also reflect mechanisms involved in the maintenance of attention. In their opinion retaining in visual short-term memory information about the silent stimuli is thought to drive top-down template signals that facilitate the maintenance of visual spatial attention, even though they are not task-relevant.

Overall, the reported results provide novel evidence pointing to the similarities between self-face and emotional face processing, i.e., similar effects are obtained in the same processing stages of these two types of stimuli. First of all, both of them capture involuntary attention (quantified by the N2pc component) as revealed by our and Grimshaw et al.'s experiments (2014). As a consequence of the above, self-face and emotional-face (Fox et al., 2002) produce attentional bias. Finally, visual short-term memory is engaged in the processing of these stimuli as revealed by the emergence of SPCN (Holmes et al., 2009). However, this highly interesting resemblance should be further investigated through direct comparison of these two types of stimuli, in order to reveal plausible common mechanisms involved in their processing.

At the end, we would like to comment on future directions in the field of the self-research. The neural basis of self-face recognition has been extensively investigated, resulting in various neuroanatomical network models (Kircher et al., 2000; Uddin et al., 2005; Platek et al., 2006) and electrophysiological correlates (Keyes et al., 2010; Tacikowski and Nowicka, 2010; Kotlewska and Nowicka, 2015) associated with processing of this unique stimulus. However, as Devue and Brédart (2011) postulate, the understanding of neural correlates of self-face recognition will not substantially improve without specifying more clearly the cognitive operations induced by self-perception. It seems that discussions in many studies in this field—including our previously published papers (Tacikowski and Nowicka, 2010; Kotlewska and Nowicka, 2015)—are mainly devoted to the description of results in light of other findings, either supporting or contradicting the currently reported ones. Unfortunately, underlying cognitive mechanisms are not sufficiently elaborated, therefore impeding and complicating the interdisciplinary research of self (Zahavi and Roepstorff, 2011). In the present study, we attempted to solve this problem by referring directly to very basic attentional mechanisms, well-documented in experimental paradigms with stimuli other than the self-face.

## Author contributions

MW and AN developed the study concept. All authors contributed to the study design. Testing and data collection were performed by all authors. MW and MN performed the data analysis and interpretation under the supervision of AN. MW and AN wrote the manuscript. All authors approved the final version of the manuscript for submission.

### Conflict of interest statement

The authors declare that the research was conducted in the absence of any commercial or financial relationships that could be construed as a potential conflict of interest.
